# Diabetic retinopathy: Involved cells, biomarkers, and treatments

**DOI:** 10.3389/fphar.2022.953691

**Published:** 2022-08-09

**Authors:** Jiahui Ren, Shuxia Zhang, Yunfeng Pan, Meiqi Jin, Jiaxin Li, Yun Luo, Xiaobo Sun, Guang Li

**Affiliations:** ^1^ Institute of Medicinal Plant Development, Peking Union Medical College and Chinese Academy of Medical Sciences, Beijing, China; ^2^ Beijing Key Laboratory of Innovative Drug Discovery of Traditional Chinese Medicine (Natural Medicine) and Translational Medicine, Beijing, China; ^3^ Key Laboratory of Bioactive Substances and Resource Utilization of Chinese Herbal Medicine, Ministry of Education, Beijing, China; ^4^ Yunnan Branch, Institute of Medicinal Plant Development, Chinese Academy of Medical Sciences and Peking Union Medical College, Jinghong, China; ^5^ Yunnan Key Laboratory of Southern Medicine Utilization, Kunming, China; ^6^ College of Pharmacy, Heilongjiang University of Chinese Medicine, Harbin, China

**Keywords:** diabetic retinopathy, cells, potential drug targets, biomarkers, drug therapy

## Abstract

Diabetic retinopathy (DR), a leading cause of vision loss and blindness worldwide, is caused by retinal neurovascular unit dysfunction, and its cellular pathology involves at least nine kinds of retinal cells, including photoreceptors, horizontal and bipolar cells, amacrine cells, retinal ganglion cells, glial cells (Müller cells, astrocytes, and microglia), endothelial cells, pericytes, and retinal pigment epithelial cells. Its mechanism is complicated and involves loss of cells, inflammatory factor production, neovascularization, and BRB impairment. However, the mechanism has not been completely elucidated. Drug treatment for DR has been gradually advancing recently. Research on potential drug targets relies upon clear information on pathogenesis and effective biomarkers. Therefore, we reviewed the recent literature on the cellular pathology and the diagnostic and prognostic biomarkers of DR in terms of blood, protein, and clinical and preclinical drug therapy (including synthesized molecules and natural molecules). This review may provide a theoretical basis for further DR research.

## 1 Introduction

Diabetic retinopathy (DR) is one of the most serious complications of diabetes mellitus and the leading cause of vision loss and blindness worldwide. The annual incidence of DR ranges from 2.2%to 12.7% due to differences in research samples across studies, such as the number of people, geographical distribution, age, and sex (Thomas R. L. et al., 2019). According to recent studies, DR is not only a diabetic microvascular complication, but also a neurodegenerative disease. Therefore, DR has recently been defined by the American Diabetes Association as a highly tissue-specific neurovascular complication of both type 1 and type 2 diabetes (Flaxel et al., 2020). In fact, the number of patients with type 2 diabetes is much higher than those with type 1 diabetes; thus, those with type 2 diabetes comprise a larger proportion of patients with DR (Flaxel et al., 2020). However, the current measure for the stages of DR is based on clinically visible retinal microvascular changes and does not include neurodegenerative lesions that might occur earlier (Abramoff et al., 2018). According to the International Council of Ophthalmology, DR can be divided into non-proliferative (NPDR) and proliferative DR (PDR), covering four overlapping stages (see Table 1 for definitions) (Wong et al., 2018; Yang et al., 2020).

**TABLE 1:** Stages of diabetic retinopathy.

Classification	Description
Mild NPDR	Microaneurysms or dot intraretinal haemorrhages occur at this stage of the disease
Moderate NPDR	As the disease progresses, hard exudates, hemorrhage spots, or “cotton wool” appear in the retina
Severe NPDR	Many more blood vessels are blocked, accompanied by the occurrence of soft exudates and hemorrhage spots
PDR	At this advanced stage, growth factors secreted by the retina trigger the proliferation of new blood vessels along with vitreous hemorrhage and fibroplasia. In addition, accompanying scar tissue can contract and cause retinal detachment

DR, diabetic retinopathy; NPDR, non-proliferative DR; PDR, proliferative DR

In the retina, retinal neurons [photoreceptors: cones and rods, horizontal and bipolar cells, amacrine cells, and retinal ganglion cells (RGCs)], glial cells (Müller cells, astrocytes, and microglia), and blood cells (endothelial cells [ECs] and pericytes, which cooperatively form the inner BRB [iBRB]) are linked to form a vital structure called the retinal neurovascular unit, and ECs and retinal pigment epithelial (RPE) cells constitute the outer blood-retina barrier (oBRB) (Yang et al., 2020; Meng et al., 2021; Tang et al., 2022). DR is caused by retinal neurovascular unit dysfunction, including loss of cells, inflammatory factor production, neovascularization, and BRB impairment (Wang and Lo, 2018), among which inflammatory process is one of the most important features (Öhman et al., 2018). A previous study showed that diabetes jeopardizes rat retinas mainly in the outer layers because the energy metabolism activity in the outer retina is vigorous and is accompanied by a large amount of metabolic waste and mitochondrial damage (He et al., 2021). The metabolic disturbances induced by DR are mainly related to metabolism of glycolysis, polyols, tricarboxylic acid, amino acids, the urea cycle, and lipids (Xuan et al., 2020). Several observational studies have shown that dyslipidemia, mitochondrial apoptosis, and oxidative stress may be the predominant pathological changes of DR (Miller et al., 2020; Fort et al., 2021; Lin et al., 2021; Rao et al., 2021). Vascular endothelial growth factor (VEGF), HbA1c, low-density lipoprotein cholesterol, myeloperoxidase, and advanced glycation end products (AGEs) are pathogenic factors of DR. The pathogenesis of DR remains unclear, and further research is yet to be conducted.

Current therapies for DR mainly include vitreoretinal surgery, laser photocoagulation, intraocular injections of anti-VEGF agents, corticosteroids, and eye drops. Among these therapies, pan-retinal photocoagulation surgery remains the primary treatment for PDR (Flaxel et al., 2020). Additionally, in recent years, stem cell replacement therapy has been evaluated for DR, although it has significant limitations (Mathew et al., 2021b). With regard to drug treatment, in the past decade, anti-VEGF drugs have had a dramatic effect on the clinical management of DR. However, clinical trials suggest that anti–VEGF agents for DR are not effective in all patients and can even bring about complications (Fu et al., 2018b). Thus, research on novel drugs based on other targets is vital for the prevention and treatment of DR.

Therefore, we reviewed the recent literature on the cellular pathology of DR. We also evaluated the diagnostic and prognostic biomarkers of DR in terms of blood, protein, and imaging techniques. Our review also included the synthetic molecules that have been used for clinical therapy and are under clinical and preclinical investigation. Finally, we reviewed the natural molecules that are under preclinical investigation to complement the current treatment for DR.

## 2 Involved cells

**FIGURE 1:**
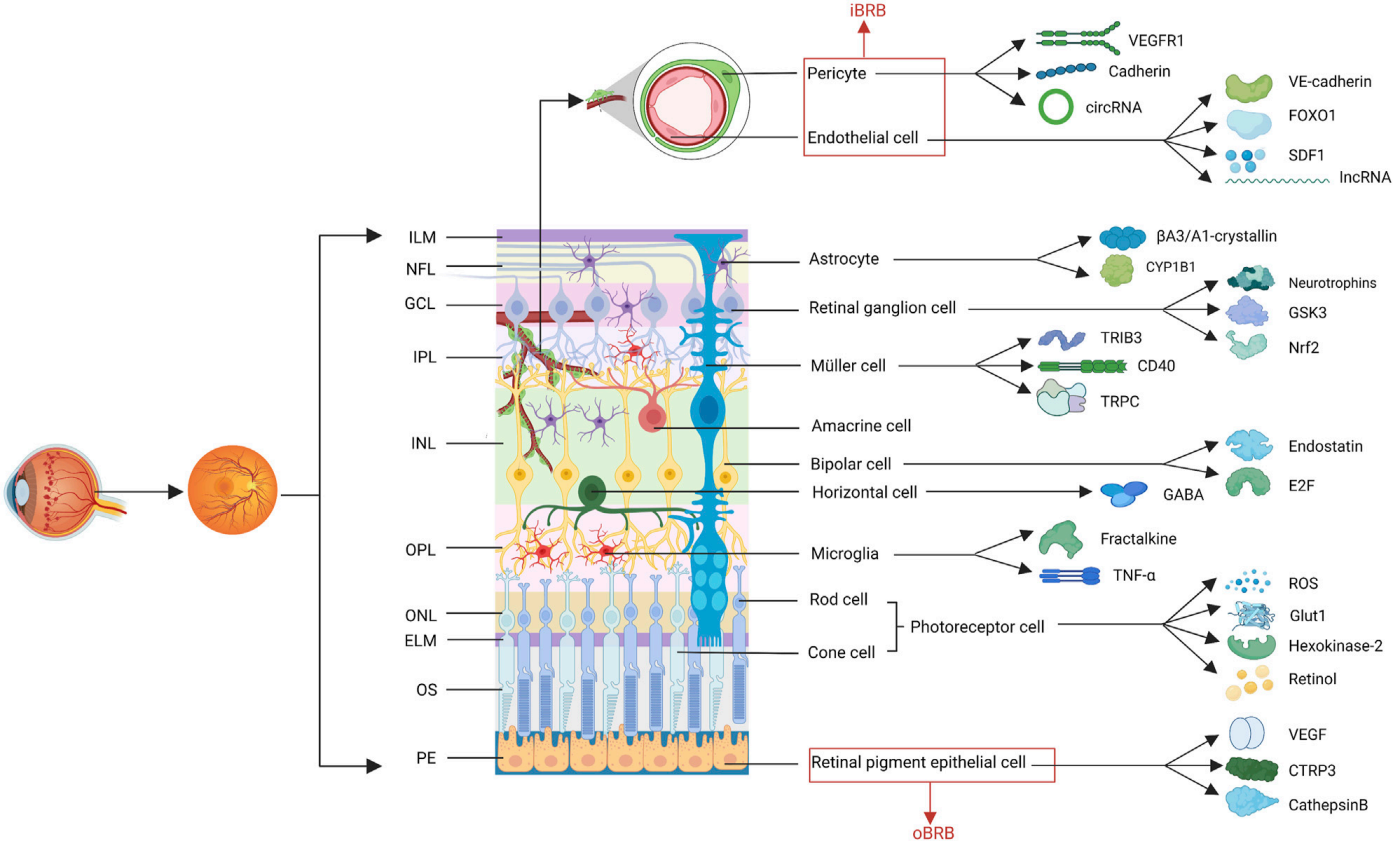
The structure of retina. The retina comprises at least 10 distinct layers, including eleven cell types involved in the progress of DR. The factors related to these cells, described in this review are shown. (ILM, internal limiting membranes; NFL, nerve fiber layer; GCL, ganglion cell layer; IPL, inner plexiform layer; INL, inner nuclear layer; OPL, outer plexiform layer; ONL, outer nuclear layer; ELM, external limiting membranes; OS, outer segments; PEL, pigment epithelium layer; iBRB, inner blood-retina barrier; oBRB, outer BRB; VEGF, vascular endothelial growth factor; VEGFR2, VEGF receptor 2; SDF1, stromal cell-derived factor 1; CYP1B1, cytochrome P450 1B1; GSK3, glucogen synthase kinase 3; Nrf2, nuclear factor erythroid 2-related factor; TRIB3, tribbles homolog 3; TRPC, transient receptor potential canonical; GABA, γ-aminobutyric acid; ROS, reactive oxygen species; Glut1, glucose transporter 1; CTRP3, C1q/TNF-related protein 3).

The retina is a highly organized tissue comprising at least 10 distinct layers (Miller et al., 2020), involving photoreceptor cells, retinal ganglion cells, bipolar cells, amacrine cells, horizontal cells, glial cells, endothelial cells, pericytes, and RPE cells (Figure 1). Diabetes causes chaos among cellular interactions and loss of almost all retinal cell populations (Szabo et al., 2017). The study of the changes of various cells in DR and the interrelationship among them can help clarify the pathological mechanism of DR and identify therapeutic targets.

### 2.1 Photoreceptor cells

Photoreceptor cells are specialized neurons in the retina that transit light into electrical signals and rely on the cycling of 11-cisretinal. The signals are transmitted to the brain for image processing (Miller et al., 2020). Photoreceptor cells have four functional regions: the outer segment, inner segment, cell body, and synaptic terminal (Tonade and Kern, 2021). The outer segments of photoreceptor cells are the main regions of energy metabolism and glycolysis, whose long-term inhibition leads to photoreceptor degeneration (Fu et al., 2018a; Fu Z. et al., 2021; Yang T. T. et al., 2021). It is worth noting that VEGF upregulation in DR is a direct contributing factor to changes in photoreceptor function (Hu et al., 2021). The two types of photoreceptor cells are rod cells, which contain rhodopsin that facilitates vision at night, and cone cells, which contain a cone pigment and are sensitive to high light. In hyperglycemia, rhodopsin expression decreases, and rod cells are more sensitive to hyperglycemia (Yang M. et al., 2021; Song et al., 2021).

Photoreceptor cells contain more than 75% of the mitochondria of the retina (Miller et al., 2020). Dysfunction in mitochondrial oxidative phosphorylation, which generates an increase in the NADH/NAD^+^ redox ratio due to a decrease in NAD^+^ regeneration and ATP deficiency, leads to photoreceptor degeneration (Mekala et al., 2019). Additionally, the OS of DR originates from the photoreceptor cells. Mitochondria are not only producers of reactive oxygen species (ROS) but also the targets of OS. A number of ROS may accumulate and damage mitochondrial DNA, which consequently reduces the expression of regulatory proteins that eliminate ROS (Bek, 2017). Sirtuin 1, a multifunctional deacetylase that is inactivated in diabetes, protects mitochondria from the activation of mitochondria-damaging matrix metalloproteinase-9 (MMP-9) and the damage of mtDNA (Mishra et al., 2018). Furthermore, active DNA methylation plays a critical role in cytosolic ROS regulation (Duraisamy et al., 2018). This vicious cycle can be affected by epigenetic machinery in the retina (Bek, 2017; Kumari et al., 2020). Until now, current certified treatments for preventing or reversing photoceptor degeneration induced by mitochondrial dysfunction have not been found.

Hexokinase-2 (HK2), a first rate-limiting isozyme in glycolysis, plays a key role in the Warburg effect exhibited by photoreceptor cells and is required for normal rod function (Weh et al., 2020). HK2 also has non-enzymatic roles in the regulation of apoptosis by interacting with mitochondria via Akt signaling (Weh et al., 2020). Therefore, it is noteworthy that the deprivation of HK2 in rods causes inhibition of retinal glycolysis and degeneration of age-related photoreceptor cells, which leads to a progressive increase in the number and size of mitochondria in the rods (Petit et al., 2018; Zhang R. et al., 2020).

In hyperglycemia, hexokinase is saturated and photoreceptor-mediated glucose metabolism is decreased, generating the accumulation of sorbitol (a kind of polyol), which is produced from excess glucose by aldose reductase via the polyol pathway (Holoman et al., 2021). Polyol accumulation is one of the key pathological features of DR that aggravates electroretinogram defects, inflammation, and OS in the retina (Holoman et al., 2021). According to these studies, reducing polyol accumulation via glucose transporter 1 (Glut1) in photoreceptor cells can be used for DR therapy (Holoman et al., 2021). Glut1 is expressed extensively in retinal cells and is thought to be the only glucose transporter in photoreceptor cells. Retinol binding protein 3, a retinol transport protein secreted primarily by photoreceptor cells, can also combine with Glut1 to restrain Müller cells and endothelial cells from taking in glucose and inducing inflammatory cytokines (Hisashi et al., 2019). Moreover, a study implied that inhibiting Glut1 expression to restrict glucose transport will decrease retinal glucose concentrations and ameliorate DR (You et al., 2017).

Another important diabetes-associated variation in photoreceptor cells is *de novo* retinol lipogenesis. The main reason for this shift is the activation of fatty acid synthase by hyperglycemia (Rajagopal et al., 2021). This shift coupled with other changes in photoreceptors may make up the early pathological mechanism of angiogenesis in early DR (Rajagopal et al., 2021). Neurovascular coupling may directly be impacted by diabetes-induced lipid perturbations at photoreceptor synapses (Nippert et al., 2018). Additionally, excess retinal saturated fatty acid can be harmful to photoreceptor energy metabolism and consequently impacts vascular coupling.

Because autophagy is an essential survival mechanism in photoreceptor cells, it plays a key role in sustaining photoreceptor function by facilitating photoreceptor outer segment degradation and visual pigment recycling (Mathew et al., 2021a; Villarejo-Zori et al., 2021). Although the mechanism by which autophagy protects photoreceptors and the relationship between DR and autophagy are ambiguous, more recent studies have corroborated the importance of autophagic flux. For example, impairment of autophagy increases superoxide formation and apoptosis in 661 W cells (a cone cell line) under hyperglycemic conditions (Taki et al., 2020).

### 2.2 Bipolar cells

Bipolar cells can accept inputs from photoreceptors and transmit signals to retinal ganglion cells (Szabo et al., 2017; Eggers and Carreon, 2020). Endostatin, an antiangiogenic protein that exists in bipolar cells, is a naturally cleaved fragment of type XVIII collagen (Bonet et al., 2021). This protein is mainly expressed in bipolar cells and photoreceptor cells. Currently, there are few reports related to bipolar cells in DR. One study has suggested that it is highly vulnerable to high glucose levels (Wang Y. et al., 2017). In diabetic mice, the appearance of intravitreal vessels was shown to be associated with a decrease in endostatin levels in the retina (Bonet et al., 2021). In addition, high sugar levels can induce the production of reactive metabolites such as methylglyoxal (MG), which results in decreased function of bipolar cells because MG is the most reactive glycation precursor of cytotoxic end products (Schlotterer et al., 2019).

In the degenerating retina of diabetic mice, some defense mechanisms against diabetes exist in bipolar cells. Functional gene networks and pathways related to inflammation (antigen processing and presentation and/or interferon response) are commonly upregulated (Van Hove et al., 2020). Moreover, the functional gene networks and pathways related to the OS response are also commonly upregulated in bipolar cells, which is reflected in phosphorylation transcripts and an increase in transcripts correlated to ATPase-mediated proton transport (Van Hove et al., 2020). In addition, E2F transcription factors play a key role in controlling cell cycle progression, and their inactivation can rescue high glucose-induced ectopic division and cell death of bipolar cells (Wang Y. et al., 2017).

### 2.3 Retinal ganglion cells

RGCs, made up of 40 subtypes, are terminally differentiated neurons in the central nervous system(Thomas et al., 2017). As efferent neurons, they transfer visual information to the brain. RGCs have a limited endogenous regenerative capacity after damage; thus, apoptosis can lead to permanent vision loss. Sphingolipid rheostat, a dynamic equilibrium consisting of ceramide, sphingosine, and sphingosine-1-phosphate, has a neuroprotective effect against excitotoxic RGC death (Nakamura et al., 2021). As the most vulnerable neurons in the retina, RGCs are the most sensitive to diabetes-induced stress reactions in the early stage of nerve growth. The excitatory toxicity of glutamate increases metabolism, the caspase cascade waterfall increases apoptosis, and other mechanisms are closely related to the damage of RGCs (Fu et al., 2020). The axonal degeneration of RGCs, which disturbs axonal transport, may be the earliest event in DR pathogenesis.

Neurotrophins, including brain-derived neurotrophic factor (BDNF), nerve growth factor (NGF), and mesencephalic astrocyte-derived neurotrophic factor (MANF), are secreted growth factors that control neuronal growth, differentiation and survival and are involved in the protection against RGC injury (Gao et al., 2017; Zhang et al., 2017). BDNF can maintain synaptic plasticity and neuronal interconnections (Behl and Kotwani, 2017). However, BDNF-mediated neuroprotective actions are downregulated during hyperglycemia. In addition, the neuroprotective effect of endogenous NGF can be hindered by pro-NGF, which enhances the expression of neurotrophin receptor p75 to stimulate RGC apoptosis in DR (Elshaer and El-Remessy, 2017; Mesentier-Louro et al., 2017; Mesentier-Louro et al., 2019). Finally, MANF is a newly discovered secreted neurotrophic factor that is highly expressed in RGCs and can protect them from hypoxia-induced injury and apoptosis by preventing endoplasmic stress-mediated apoptosis *in vivo* and *in vitro* (Gao et al., 2017).

In high-fat diet -induced DR, tau hyperphosphorylation causes vision deficits and synapse loss in RGCs by destabilizing microtubule tracks, damaging microtubule-dependent synaptic targeting of cargo such as mRNA and mitochondria, and disrupting synaptic energy production in mitochondria via glucogen synthase kinase 3 (GSK3) activation (Zhu et al., 2018). In high-fat diet-induced DR mouse models, mitochondrial degeneration occurs when downregulation of β-catenin activation due to abnormal activation of GSK3β causes synaptic neurodegeneration of RGCs by suppressing ROS-scavenging enzymes, thus triggering OS-driven mitochondrial impairment (Shu XS et al., 2020).

OS is closely associated with RGC apoptosis. As a neuroprotective protein, transforming growth factor-β (TGF-β) overregulates hyperglycemia to protect RGCs from harm by activating stress response proteins and antioxidant pathways, such as aldehyde dehydrogenase 3A1, heme oxygenase-1 (HO-1), hypoxia-inducible factor (HIF) -1α, and nuclear factor erythroid 2-related factor (Nrf2) (Chen et al., 2020). Among these proteins, Nrf2 is a pivotal nuclear transcription factor that protects cells against oxidative injury. Normally, Nrf2 combines with Kelch-like erythroid-cell-derived protein with CNC homology-associated protein 1 (Keap1) as a complex to be the target of proteasomal degradation, and Nrf2 transcriptional activity is decreased in the wake of increased Keap1 levels (Radhakrishnan, 2020). Long non-coding RNA (lncRNA) is important in modulating the expression of Nrf2 in DR. The Sox2 overlapping transcript, a type of lncRNA, is a key regulator of oxidative stress in RGC damage of patients with DR (Zhang et al., 2017). Sox2 overlapping transcript knockdown can lead to the accumulation of Nrf2 protein and nuclear translocalization to combat oxidative stress by intercepting Nrf2/Keap1 contact in RGCs and activating Nrf2 and HO-1 signaling (Li et al., 2017; Liu X.-F. et al., 2018). Studies have shown that overexpression of sulfiredoxin 1 and senescence marker protein 30 may protect RGCs from high glucose (HG)-induced injury by enhancing Nrf2 expression via regulation of the Akt/glycogen synthase kinase-3β axis (Zhu F. et al., 2021; Zhang et al., 2021). Sulfiredoxin 1 is a member of the endogenous antioxidant sulfiredoxin protein family, while senescence marker protein 30 is an aging-related protein. Another important antioxidant mechanism in RGCs is the downregulation of the pleckstrin homology domain and leucine rich repeat protein phosphatase 1, which activates Nrf2/antioxidant response element -mediated transcription (Zhang X. et al., 2020). Furthermore, in high glucose treatment, Brahma-related gene 1 expression is significantly downregulated, also leading to a decline in Nrf2/HO-1 signaling (Sun et al., 2020).

### 2.4 Amacrine cells

Amacrine cells, made up of approximately 40 different cell types, are characterized by a wide variety of shapes, sizes, and stratification patterns, which are still under investigation. In diabetes, the immunoreactivity of AII amacrine cells appears to decrease and change, and a patchy appearance of AII amacrine cell degeneration can be observed (Szabo et al., 2017). The loss of cholinergic amacrine cells, known as starburst amacrine cells, may cause a serious decrease in the optokinetic response (Baya Mdzomba et al., 2020). In addition, dopaminergic amacrine cells degenerate in diabetic rat retinas, as revealed by transferase-mediated dUTP nick-end labeling staining (Ma et al., 2018). According to the electroretinography of oscillatory potentials, in the very early changes of the diabetic retina, the relevance of the interrelations between vascular and functional elements mainly involves the precocious involvement of amacrine cells in diabetic eyes, because amacrine cells specifically modulate the regulation in the middle retina (Midena et al., 2021a).

### 2.5 Horizontal cells

In horizontal cells, glucose transport into photoreceptor synapses relies on glucose transporter-2 (Yang M. et al., 2021). In a HG environment, γ-aminobutyric acid (GABA) immunoreactivity (IR) is increased in horizontal cells by decreasing GABA transporter (GAT)-1-IR and increasing GAT-3-IR to induce GABA accumulation (Carpi-Santos et al., 2017). Normally, GABA accumulation affects cytoactivity; however, in DR organotypic retinal models, HG did not affect horizontal cell viability (Yang M. et al., 2021). There are few studies on the pathological changes in horizontal cells in DR, and further tests are needed.

### 2.6 Glial cells

Glial cells are supporting cells of the neural retina and play an important role in the immune system of the retina (Rubsam et al., 2018). Diabetes can cause homeostatic changes in the retina that affect these glial cells; thus, glial cells promote inflammation in the retina, which is a driving force for sustaining angiogenesis in PDR (Rezzola et al., 2017). These cells can be divided into three major categories: two types of macroglia (Müller cells and astrocytes) and microglia. Microglia and astrocytes are the main sources of ROS in central nervous system chronic degenerative diseases (Mendonca et al., 2020).

#### 2.6.1 Müller cells

Müller cells, representing 90% of retinal glia, are the main types of glial elements. They provide the structural, metabolic, and neurotrophic support necessary for the retinal layers. Endoplasmic reticulum (ER) stress in Müller cells is directly linked to retinal inflammation in DR (Yang et al., 2019). X-box binding protein 1, an effector of the unfolded protein response activated by inositol-requiring enzyme 1α, is a major transcription factor that regulates ER chaperones and ER-associated degradation (Yang et al., 2019). The activation of nucleotide-binding domain-like receptor protein-3 (NLRP3) inflammasome-mediated inflammation in Müller cells can result in BRB damage (Wei et al., 2021). Additionally, overexpression of tribbles homolog 3 (TRIB3) protein, a regulator of insulin signaling in diabetes, can reduce Müller cell viability (Pitale et al., 2021). TRIB3 is a major regulator of diabetic retinal pathophysiology and is upstream of HIF-1α, epidermal growth factor receptor, and glial fibrillary acidic protein. TRIB3 controls glucose metabolism, cytokine expression, and gliosis in retinal cells via HIF-1α-mediated Glut1 and epidermal growth factor receptor expression (Pitale et al., 2021).

Circular junctions exist between photoreceptors and Müller cells. Retinol binding protein 3, which is secreted by photoreceptors, can inhibit glucose uptake into Müller cells, leading to the decreased expression of VEGF and interleukin (IL) –6 (YokomizO et al., 2019). VEGF localizes to glial cells of the inner retina and anterior optic nerve and is expressed in the retinas and optic nerves of diabetics before retinal neovascularization (Mrugacz et al., 2021). In addition, Müller glia-derived exosomes can promote angiogenesis in DR. For example, exosome miR–9–3p promotes angiogenesis by restricting sphingosine–1-phosphate receptor (Liu et al., 2022).

Many factors can promote the inflammatory response of Müller cells, including hyperglycemia, elevated HIF–1, insulin-like growth factor 1, and CD40. HIF–1 and insulin-like growth factor 1 in the serum and vitreous body of diabetic patients activate Müller cells to form a chronic inflammatory milieu (Gaonkar et al., 2020). CD40 is an immune co-stimulatory molecule that plays a key role in Müller cells. CD40 activation can induce ATP release in Müller cells, resulting in the activation of P2X7 purinergic receptors on retinal microglia and their subsequent expression of inflammatory cytokines (Portillo et al., 2017). Furthermore, Müller cells first produce an inflammatory response in the diabetic retina andactivate signal microglia (Abcouwer, 2017).

In a study, transient receptor potential canonical (TRPC) channel, a cation channel of the transient receptor potential family, was reported to have a predominant action in Müller cells and microglia and was found to be expressed in mouse retinas in large quantities (Sachdeva et al., 2018). TRPC-mediated processes aggravating retinal neurodegeneration and vasoregression may be due to a TRPC-mediated accumulation of the reactive metabolite MG and its detoxification by Glyoxalase 1 (Sachdeva et al., 2018). However, the exact TRPC-dependent processes in DR need to be identified to understand the precise causative role of TRPCs (TRPC1-TRPC7) in DR.

#### 2.6.2 Astrocytes

Astrocytes, which are named after their stellate shape, are located in the innermost retinal layers, and serve as a link between the retinal blood vessels and neurons. Astrocytes are also crucial for maintaining the normal function of BRB (Fresta et al., 2020b). Waves of cytosolic calcium (Ca^2+^) are vital for maintaining glia–astrocyte, astrocyte–astrocyte, and astrocyte–neuron communication (Shahulhameed et al., 2019). In diabetes, astrocytes are upregulated at the mRNA level and secrete various pro-inflammatory cytokines, such as IL-1β and IL-6 to amplify the inflammatory response (Rubsam et al., 2018). Hyperglycemia also significantly enhances the expression of pro-oxidants (iNOS, Nox2), nuclear translocation, activation of pNF-κB and Nrf2, and secretion of HO-1 in astrocytes (Fresta et al., 2020b).

As a lens protein expressed in astrocytes, βA3/A1-crystallin is an uncompetitive inhibitor of human protein tyrosine phosphatase 1B (PTP1B) enzyme, whereas PTP1B controls signal transducer and activator of transcription 3 phosphorylation to regulate the expression of βA3/A1-crystallin genes in astrocytes. PTP1B4 is an enzyme that links glucose metabolism and inflammation in diabetes and has a positive association with vitreous humor levels of VEGF, IL-8, and monocyte chemoattractant protein 1. Therefore, βA1-crystallin/PTP1B signaling may regulate inflammatory signaling in astrocytes under hyperglycemic stress. Moreover, βA1-crystallin is the dominant isoform of glucose metabolism in astrocytes and is essential for maintaining mitochondrial function and oxidative stress during HG stress (Ghosh et al., 2021).

Additionally, cytochrome P450 1B1 belongs to the family of heme-containing terminal oxidases, CYP450s, and engages in metabolic activation and detoxification of many compounds. The expression and activity of cytochrome P450 1B1 play an important role in resisting DR by modulating astrocytes proliferation, migration, and morphogenesis, and forming a proper fibronectin network to support retinal neurovascular function (Falero-Perez et al., 2019).

#### 2.6.3 Microglia

Microglial cells are mononuclear phagocytes that can be regarded as tissue-resident macrophages in the retina. Microglia are also the major cell population in vitreous fibrovascular membranes. GPNMB^+^ (glycoprotein nonmetastatic melanoma protein B) microglia, a subpopulation of vitreous fibrovascular membranes, exhibit both profibrotic and fibrogenic properties. The profibrotic microglia and cytokines upregulated in vitreous PDR exhibit ligand-receptor interactions (Xiao et al., 2021). Additionally, the microglial population and complement system form immune defense mechanisms in the retina. During early to late changes in DR, microglia can mediate activation of the alternative complement pathway based on complement factor H and CD11b (integrin αM) gene expression to resist inflammation (Shahulhameed et al., 2020).

Microglia contact neuronal synapses and capillaries in the inner retina. There are two distinct microglial populations in the oxygen-induced retinopathy retina: activated-amoeboid phenotype microglia reside on the surface of the superficial capillary plexus of the retina, whereas resting ramified morphology microglia reside in the deep capillary plexuses (Uddin et al., 2020). Microglia can modulate vessel diameter and express vasoactive genes, likely via modulation of the local renin-angiotensin system (Mills et al., 2021). Under normal physiological conditions, retinal cells potentially restrain excessive microglial activation by secreting anti-inflammatory factors, including TGF-β2 secreted by the cones (Xiao et al., 2021). In diabetes, microglial cell morphology transitions from a resting-ramified morphology to an activated-amoeboid phenotype and inflammation-related cytokines are released (Chang et al., 2019). The activation of microglia within the PDR microenvironment involves several pathways, including interferon gamma receptor 1, chemotactic cytokine receptor 5, and CD44 signaling (Xiao et al., 2021). When cytokine-mediated inflammatory responses are highly expressed in the retina, the proliferation of microglia is increased, which shifts from an anti-inflammatory to a pro-inflammatory state (Szabo et al., 2017). In an experimental model of diabetes, activated microglial cells penetrated the basement membrane of the inner BRB (iBRB) and engulfed endothelial cells, leading to an increased number of acellular capillaries and albumin leakage, which is a significant factor in iBRB breakdown (Xie et al., 2021). Such damage to the iBRB was recently found to be preventable by inhibiting colony-stimulating factor 1-receptor (Kokona et al., 2018).

In DR, many factors are associated with the excessive activation of microglia. Fractalkine (FKN), a chemokine expressed constitutively by healthy neurons, signals microglia upon interaction with the CX3CR1 receptor. The expression of FKN declines with diabetes progression and activates microglia in the retina, resulting in an increase in IL-1β expression in microglia and astrocytes, fibrinogen deposition, and perivascular clustering of microglia (Mendiola et al., 2016; Jiang et al., 2022). Furthermore, FKN/CX3CR1 is a key signaling pathway in inducing capillary constriction, relying on microglial contact and FKN-CX3CR1-mediated up-regulation of angiotensinogen (Mills et al., 2021). MG-derived AGEs play an important role in activating microglia and accumulate in neuronal compartments of the retina during hyperglycemia (Schlotterer et al., 2019). Microglial activation is also mediated by autocrine pro-inflammatory factors, such as tumor necrosis factor-α (TNF-α) (Xiao et al., 2021). Moreover, TRIB3 mediates the increase in retinal microglia and the expression of VEGF, NF-κB, and other cytokines, thus modifying the early inflammatory response in hyperglycemia (Pitale et al., 2021).

Many inflammation-associated cytokines are expressed in microglia, including HIF-1α (Uddin et al., 2020), IL-8, TNF-α, MMP9. Pro-inflammatory markers IL-8 and MMP9 are primarily secreted from microglial cells, resulting in significant upregulation of platelet-endothelial cell adhesion molecule and VEGF-VEGF receptor 2 (VEGFR2) binding in the PDR vitreous (Shahulhameed et al., 2020). In a diabetic mouse model, the accumulation of aldose reductase accelerated VEGF protein expression and amadori-glycated albumin -induced TNF-α secretion and cell migration (Chang et al., 2019). Microglial cells are a major source of TNF-α (Xiao et al., 2021). Additionally, Nogo-A modulates phosphorylation signaling, which can increase TNF-α secretion in microglia (Baya Mdzomba et al., 2020). Nogo-A is endogenously expressed in Müller cells and RGCs. Moreover, Nogo-A is upregulated in the retina of patients with DR, and may be released into the vitreous stem from RGC lysis. Finally, hyperglycemia-induced expression of NF-κB signaling is also related to the secretion of TNF-α (Xiao et al., 2021).

### 2.7 Pericytes

Pericytes are one of the types of cell that form the iBRB. Pericytes envelop the microvasculature, adhere to the abluminal surface of endothelial tubules, and are the first vascular cells affected by diabetes. The key adherens junction protein between the endothelium and pericytes is N-cadherin (Monickaraj et al., 2018). Pericyte/endothelial cell interaction is affected by Ephrin-B2 which is overexpressed in diabetes (Coucha et al., 2020). Pericyte functions in the retina include promoting endothelial sprouting, expressing VEGFR1 by pericytes, spatially restricting VEGF signaling, and maintaining the integrity of the BRB (Eilken et al., 2017). Pericytes are vital to the BRB because platelet-derived growth factor (PDGF)-B/PDGF receptor beta (PDGFRb) signaling is significant in the formation and maturation of BRB via the active recruitment of pericytes to growing retinal vessels (Park et al., 2017). PDGF-B is also an indispensable factor required for pericyte survival (Monickaraj et al., 2018). In addition, pericyte maturation, changing from a stellate shape and high proliferation to quiescent and elongated states, is necessary for vessel remodeling during angiogenesis (Figueiredo et al., 2020).

Circular RNAs (circRNAs) such as cZNF532 and cPWWP2A play an important role in regulating retinal pericyte degeneration and vascular dysfunction. For example, cZNF532 is principally produced in the cytoplasm of pericytes, and its overexpression reduces the diabetic effect on microangiopathy by acting as an miR-29a-3p sponge and inducing expression of NG2, LOXL2, and CDK2. Meanwhile, cPWWP2A acts as an miR-579 sponge that can contribute to the promotion of DR progression through upregulation of the angiopoietin-1/Occluding/Sirtuin 1 proteins (Liu C. et al., 2019; Jiang et al., 2020). Moreover, hypoxia-induced circEhmt1 in the nucleus of pericytes is a kind of circRNA that can be transferred from pericytes to endotheliocytes by exosomes, playing an important role in regulating pericyte-endotheliocyte crosstalk by mediating the NFIA/NLRP3 pathway to activate HIF signaling (Ye et al., 2021).

In early diabetic models, the total number of pericytes remained static, varying by the distribution of on-vessel versus off-vessel pericytes to form the pericyte bridge. The combination of pericytes and microvasculature is a dynamic process, and pericytes clearly move off the vessel and frequently extend and retract filopodia between vessels in time-lapse imaging of lineage-marked pericytes (Corliss et al., 2020). Pericyte detachment is a key mechanism underlying bridge cell and basement membrane bridge formation and is an essential factor that accelerates DR progression, further destabilizing retinal vascular endothelial cells (Park et al., 2017; Corliss et al., 2020). AGEs can activate Rho-kinase to mediate moesin phosphorylation at Thr558, and the resulting phospho-moesin interacts with CD44 to form the CD44 cluster, which may stimulate the migration of pericytes and subsequent pericyte detachment in microvessels (Zhang S. S. et al., 2020).

With the development of diabetic retinopathy, the loss of pericytes occurs in the retina, which causes vascular leakage due to inadequate pericyte coverage, leading to the eventual destruction of the microvasculature, while BRB permeability is increased facilitating low-density lipoprotein penetration into the retina (Park et al., 2017). Various factors influence pericyte loss through different pathways. For example, hyperglycemia-induced ER stress may lead to apoptosis and pericytes loss (Lai et al., 2017). High levels of thyroid stimulating hormone may facilitate the effect of high glucose-induced pericyte loss through thyroid stimulating hormone -receptor -dependent mitochondrial apoptosis in retinal pericytes (Lin et al., 2021). DNA methyltransferase-1 can prevent the overexpression of peroxisome proliferator-activated receptor α by mediating its methylation to increase apoptotic cells and ROS in pericytes (Zhu Y. et al., 2021). Overexpression of soluble epoxide hydrolase can initiate the loss of pericytes by generating diol 19,20-dihydroxydocosapentaenoic acid (19,20-DHDP) from docosahexaenoic acid in diabetic retina (Hu et al., 2017). Mechanistically, 19,20-DHDP can induce VE-cadherin internalization and the migration of vascular pericytes into the extravascular space, decrease N-cadherin expression, and reduce the association of cholesterol with PS1-VE-cadherin and PS1-N-cadherin complexes (Hu et al., 2017). Increased levels of cathepsin D, an aspartyl protease, can alter endothelial-pericyte interactions by decreasing N-cadherin and PDGFRb, increasing the phosphorylation of the downstream signaling protein, protein kinase C-alpha, and Ca^2+^/calmodulin-dependent protein kinase II (Monickaraj et al., 2018). Recent studies in C57BL/6 diabetic mouse retinas have indicated that TRIB3 can affect the formation of acellular capillaries and subsequent pericyte loss (Pitale et al., 2021). Excessive autophagy also causes stress and pericyte necrosis (Mao et al., 2017).

### 2.8 Endothelial cells

ECs are another main cell type found in the iBRB that form a smooth internal vascular lining and control the exchange of chemical substances between the blood and the retina. Endothelial tip cells are specialized ECs, and their ability to invade and migrate into tissues can influence sprouting angiogenesis (Figueiredo et al., 2021). The invasiveness of endothelial tip cells during angiogenesis depends on the formation of specific actin-related protein 2/3 -dependent dactylopodia, which are derived from filopodia. Actin-related protein 2/3 activation can be restricted by myosin IIA by positively regulating the maturation state of focal adhesions and negatively regulating the β-PIX/Rac1 pathway (Figueiredo et al., 2021).

In hyperglycemia, the endothelial markers CD31 and VE-cadherin are decreased in ECs, and VE-cadherin disruption leads to the loss of cell-to-cell contact in EC monolayers and mediates Sema4D/PlexinB1 to induce EC dysfunction (Portillo et al., 2017; Thomas A. A. et al., 2019; Wu et al., 2020). The increased expression of the mesenchymal markers α-smooth muscle actin, smooth muscle 22, fibroblast-specific protein 1, and vimentin in ECs demonstrates that damaged ECs transform into a mesenchymal phenotype, termed endothelial-mesenchymal transition (EMT) (Thomas A. A. et al., 2019).

As pericytes are lost, tube formation by ECs increases, which is related to endothelial angiogenesis (Ye et al., 2021)**.** Pericyte depletion can induce forkhead transcription factor FOXO1 activation in unstable ECs, possibly through reduced Tie2-mediated PI3 kinase/Akt signaling and upregulation of angiopoietin-2, which contributes to the elevation of vascular destabilizing factors including Ang2 and VEGFR2, leading to vessels susceptible to leakage upon external VEGF-A stimulus (Park et al., 2017). Notably, pericyte loss directly leads to inflammatory responses in ECs and perivascular infiltration of macrophages, whereby macrophage-derived VEGF and placenta growth factor can also activate VEGFR2 in ECs (Ogura et al., 2017). Excess adiponectin can decrease vascular barrier function by increasing adhesion molecule-1 in ECs, enhancing VEGF-VEGFR2 signaling, and inducing EC migration via AdipoR1 and AdipoR2 pathways to mediate angiogenesis (Nishinaka et al., 2021). The hyperglycemia-mediated increase of EC-derived adhesion molecule–1 is particularly responsive to the downregulation of Nox1/4/5, which is the Nox isoform present in ECs that produces excess ROS and is overexpressed to enhance EC proliferation and tubule formation in DR (Deliyanti et al., 2020). Because of the intimate connection between pericytes and ECs, factors that impact the loss of pericytes also play a role in the loss of ECs in DR, which leads to the formation of acellular capillaries such as 19,20-DHDP and TRIB3 (Lai et al., 2017). Then, 19,20-DHDP disrupts VE-cadherin continuity in cultured ECs and increases EC permeability to dextran (Hu et al., 2017). TRIB3, which is expressed in ECs of the human diabetic retina and controls the death of ECs, has been linked to aberrant angiogenesis. In C57BL/6 diabetic mouse retinas, TRIB3 upregulates adhesion molecule-1, a cell surface glycoprotein expressed in ECs (Pitale et al., 2021).

ER stress also promotes endothelial dysfunction in DR, and the predominant inducer is chemokine SDF1 (stromal cell-derived factor), also known as CXCL12 (Lai et al., 2017). The level of SDF1α, a subtype of SDF1, is positively correlated with Nε-(carboxymethyl) lysine, a major AGE, and its expression is significantly increased in patients with diabetes. Nε-(carboxymethyl) lysine can cause retinal EC dysfunction and vascular leakage through a therapeutic targeting tumor progression locus-2/activating transcription factor-4/SDF1α-signaling pathway in DR (Lai et al., 2017).

Recent DR research has focused on lncRNAs as key regulators of EC function. For example, vascular endothelial-associated lncRNA VEAL2 competitively restrains the overactivation of protein kinase C beta 2 (PRKCB2) in ECs by binding to the DAG-binding domain to rescue the effects of PRKCB2-mediated turnover of endothelial junctional proteins, thus maintaining normal endothelial permeability (Sehgal et al., 2021). The lncRNA, MALAT1 can downregulate its expression through siRNAs to prevent a glucose-induced increase in Keap1 and modulate the transcriptional activity of Nrf2 to maintain the antioxidant defense system in DR (Radhakrishnan, 2020; Radhakrishnan and Kowluru, 2020). Both H19 and SNHG7 can prevent EMT. Overexpression of H19 restrains EMT via TGF-β1 and suppresses TGF-β signaling by intercepting the MAPK–ERK1/2 pathway, whereas SNHG7 acts via the miR-34a-5p/XBP1 axis (Thomas A. A. et al., 2019; Cao et al., 2021). However, some lncRNAs accelerate the progression of DR, such asTDRG1, which promotes microvascular cell dysfunction by mediating VEGF overexpression (Gong et al., 2019).

Additionally, circRNAs, such as circHIPK3, circCOL1A2, and circFTO are potential targets for controlling PDR and play an important role in retinal vascular dysfunction. For example, circHIPK3 increases endothelial proliferation by blocking miR-30a function (Shan et al., 2017). Moreover, circCOL1A2 and circFTO can facilitate angiogenesis during the pathological progression of DR, and their respective pathways are the miR-29b/VEGF axis and the miR-128–3p/thioredoxin-interacting protein axis (Zou et al., 2020; Guo et al., 2021). Finally, the roles of miR-29b and miR-128–3p suggest that microRNA is also significant for angiogenesis in DR.

### 2.9 Retinal pigment epithelial cells

The monolayer RPE cells constitute the oBRB and locate between the neuroretina and choroid. The interaction between RPE cells and microglia affects the integrity of the oBRB in DR (Jo et al., 2019). In addition, the apical microvilli of RPE cells wrap around OS of adjacent photoreceptors and provide nutritional support to photoreceptors (Mekala et al., 2019; Keeling et al., 2020).

VEGF is a vital factor for promoting angiogenesis, and its secretion in RPE cells is regulated by L-type calcium channels and pituitary adenylate cyclase-activating polypeptide (Fabian et al., 2019; Vuori et al., 2019). Pituitary adenylate cyclase-activating polypeptide is a peptide with a wide range of functions that can attenuate the levels of VEGF, endothelin-1, and angiogenin in RPE cells (Fabian et al., 2019).

HG levels can promote the migration and proliferation of RPE cells, reducing the expression of epithelial markers E-cadherin and ZO-1 and increasing the levels of mesenchymal markers vimentin and α-SMA, indicating EMT (Yang J. et al., 2021). A recent study showed that knockdown of miR-195 can inhibit EMT and RPE cell permeability (Fu S. H. et al., 2021). In addition, hyperglycemia can stimulate the production of ROS, increase the level of malondialdehyde, and reduce SOD activity in RPE cells (Zhang and He, 2019). It is worth noting that C1q/TNF-related protein 3 (CTRP3) and miR-455–5p may be new therapeutic targets for oxidative stress and apoptosis in RPE cells (Chen et al., 2019; Zhang and He, 2019). The Nrf2/HO–1 pathway is a target of CTRP3, and suppressor of cytokine signaling 3 is a direct target of miR-455–5p (Chen et al., 2019; Zhang and He, 2019). Furthermore, CTRP3 mediates iBRB compatibility and resists vascular permeability induced by DR through the AMPK-dependent occludin/claudin –5 signaling pathway (Yan et al., 2022).

Lysosome membrane permeabilization is induced in RPE cells under diabetic conditions, which leads to massive amounts of cathepsin B being released from the lysosomes into the cytosol in RPE cells under HG conditions and subsequent autophagy-lysosome pathway dysfunction (Patel et al., 2018; Feng et al., 2021). The expression of lysosome membrane permeabilization can be upregulated by overexpressed high mobility group box, a nuclear DNA-binding protein with various functions, via a cathepsin B-dependent pathway (Feng et al., 2021). Additionally, lysosome membrane permeabilization may initiate mitochondrial membrane potential changes by interacting with BCL2 family members, CYCS (cytochrome c, somatic), and ROS release, which activates the classical mitochondria-caspase pathway (Feng et al., 2021).

## 3 Biomarkers

The latest definition of biomarker is “a defined characteristic that is measured as an indicator of normal biological processes, pathogenic processes, or responses to an exposure or intervention, including therapeutic interventions” according to the FDA-NIH Joint Leadership Council. Sensitive, accurate, and specific detection of biomarkers is important for diagnosing and measuring the severity of DR, and provides a powerful and dynamic approach to improve our understanding of the mechanisms underlying DR (Tamhane et al., 2019). The discovery of novel biomarkers for detecting DR remains vital.

### 3.1 Blood

Blood is the primary source of biomarkers for DR detection. Blood metabolic biomarkers include proteins, glycoproteins, polypeptides, and amino acids, of which proteins are the most prevalent. Currently, HbA1C is the only validated systemic biomarker in DR and has been used in clinical diagnosis. Most of the research on other biomarkers is still in the preclinical basic research stage, and the more studied biomarkers of DR-angiogenesis (e.g., VEGF), inflammatory factors (e.g., IL-6, IL-8, IL-1β, IL-17A, and TNF-α), oxidative stress products (e.g., lipoperoxides and malondialdehyde), antioxidants (e.g., glutathione, glutathione peroxidase, and SOD), and apoptosis factors (e.g., Cytochrome-C) have been summarized in a number of reviews. Therefore, we have listed some of the potential biomarkers of DR that have been studied in the last 5 years (Table 2). Furthermore, individual biomarkers as well as panels of biomarkers can be used in clinical practice. In fact, the sensitivity of combinatorial biomarkers is significantly higher than that of individual biomarkers. For example, a biomarker panel consisting of 12-hydroxyeicosatetraenoic acid and 2-piperidone exhibited a faster and more accurate performance than HbA1c in diagnosing DR *in vitro* (Xuan et al., 2020).

**TABLE 2:** Blood metabolic biomarkers.

Variety	Biomarker	Role	Relevance	References
Protein	ADAMs	Angiogenesis promotion	Positive	Opdenakker and Abu El-Asrar, (2019)
	ANGPTL3	Enhance EC adhesion and migration	Positive	Yu et al. (2018)
	AOC3	Induce oxidative stress, AGEs, and oxidation of low-density lipoproteins; promote inflammation	Positive	Tekus et al. (2021)
	CTRP3	Mitigate retinal vascular permeability	Negative	Yan et al. (2022)
	FABP4	Angiogenesis promotion	Positive	Zhang et al. (2018)
	iNOS	Promote inflammation	Positive	He et al. (2021)
	Lp-PLA2	Promote inflammation	Positive	Siddiqui et al. (2018)
	MMPs	Angiogenesis promotion	Positive	Opdenakker and Abu El-Asrar, (2019)
	PTX3	Modulate inflammation and inhibit angiogenesis	Positive	Stravalaci et al. (2021)
	TGF-β	Promote inflammation	Positive	Bonfiglio et al. (2020)
	TIMPs	Affect angiogenesis and cell migration	Positive	Opdenakker and Abu El-Asrar, (2019)
Glycoprotein	PEDF	Downregulate the angiogenic, fibrogenic, and proinflammatory factors	Negative	Cheung et al. (2019)
Amino acid	Homocysteine	Induce angiogenesis, ER stress, oxi, and epigenetic modifications	Positive	(Malaguarnera et al., 2014; TAWFIK et al., 2019)
Fatty acid	17(RS)-10-*epi*-SC-∆^15^–11-dihomo-IsoF	Derivative from adrenic acid oxidation	Positive	Torres-Cuevas et al. (2021)

ADAMs, a disintegrin and metalloproteinases; ANGPTL3, angiopoietin-like 3; EC, endothelial cells; AOC3, amine oxidase copper containing 3; CTRP3, C1q/TNF-related protein 3; FABP4, fatty acid-binding protein 4; iNOS, nitric oxide synthase; Lp-PLA2, lipoprotein-associated phospholipase A2; MMPs, matrix metalloproteinases; PTX3, long pentraxin 3; TGF-β, transforming growth factor-β; TIMPs, tissue inhibitors of metalloproteases; AGEs, advanced glycation end products; PEDF, Pigment epithelium-derived factor; ER, endoplasmic reticulum.

The usual techniques for testing include immunoassay, western blot analysis, enzyme-linked immunosorbent assay, and radical absorbance capacity assay. However, the abundance of some biomarkers is low, requiring more sensitive techniques with lower detection limits. For example, the newly developed optoelectrokinetic bead-based immunosensing can detect the low-abundance biomarker of DR, lipocalin 1 (Wang J. C. et al., 2017). Additionally, to achieve more accurate and precise plasma quantification of biomarkers for patients with DR, high-throughput tools such as the integrated plasma proteome sample preparation system should be developed (Li et al., 2021).

Vitreous humor (VH), aqueous humor (AH), and tears can also be used to detect biomarkers. Compared to other body fluids, the collection of tears is easy and noninvasive, although there is currently no entirely efficient and universal method for its collection. More importantly, biomarker levels, such as those of TNF-α and VEGF in tears are comparable to those in the blood and increase with the severity of the disease (Nandi et al., 2021). However, tears mainly provide exact information regarding disorders in the anterior segment. For posterior segment disorders, AH and VH are the most suitable matrices for evaluating relevant biomarkers but their collection is invasive and difficult, which can cause secondary damage in diseased eyes (Tamhane et al., 2019).

### 3.2 Nucleic acids

Through immunohistochemistry, PCR, western blot analysis, high-throughput sequencing, Gene Ontology enrichment analysis, Kyoto Encyclopedia of Genes and Genomes pathway analysis, and other techniques, nucleic acid biomarkers related to DR can be explored. Several epigenetic modifications have been studied in DR, including methylation of DNA molecules, chromatin remodeling, histone modification, and non-coding RNA (ncRNA). These alterations can be prognostic, therapeutic, or diagnostic biomarkers of DR (Table 3) (Lopez-Contreras et al., 2020). Moreover, ncRNAs, including miRNAs, lncRNAs, and circRNAs, are the most studied biomarkers of DR. NcRNAs can be secreted into the body fluid to play a role in the pathomechanism of DR, and its primary pathway is executed in exosomes. Recently, exosomal nucleic acids, in particular, exosomes and their ncRNA payloads, have attracted much attention, as they may not only serve as specific biomarkers in the diagnosis of DR but also as promising therapeutic agents for the treatment of DR (Liu et al., 2020; Liu et al., 2022). In addition, ncRNAs can interact to regulate the progression of DR. For example, SNHG4 may sponge miR-200b by upregulating oxidation resistance 1, thus suppressing RPE cell apoptosis (Yu J. et al., 2021).

**TABLE 3:** Nucleic acid biomarkers.

Profile	Biomarker	Role	Relevance	References
DNA	DMSs in S100A13	Epigenetic biomarkers	Positive	Li et al. (2020a)
	Atg16L1	Related to autophagy	Positive	Gao et al. (2021)
miRNA	miR-1281	Microvascular promotion	Positive	Greco et al. (2020)
	miR-431–5p	Proliferation of ECs	Positive	Yu et al. (2021a)
	miR-9-3p	Angiogenesis promotion	Positive	Liu et al. (2022)
	miR-29b	Anti-apoptotic and antifibrotic effects	Negative	Dantas da Costa et al. (2019)
	miR-200b	Apoptosis promotion	Positive	Dantas da Costa et al., 2019; Yu et al., 2021c)
	miR-146a-5p	Anti-inflammatory and vascular protection		Barutta et al. (2021)
lncRNA	SNHG4	Inhibition of apoptosis	Negative	Yu et al. (2021c)
	PVT1	Promote the proliferation and migration of ECs	Positive	Wang et al. (2022)
circRNA	circ-PSNE1	Promote ferroptosis of RPE cells	Positive	Zhu et al. (2021c)
oxidative DNA breakdown product	8-OHdG	Related to oxidative stress	Positive	Hainsworth et al. (2020)

SNHG4, small nucleolar RNA, host gene 4; 8-OHdG, 8-hydroxy-2 -deoxyguanosine; RPE, retinal pigment epithelial.

### 3.3 Imaging techniques

Imaging techniques are mainly used for the diagnosis of microangiopathy in DR, which can detect and stage DR. Slit-lamp biomicroscopy, fundus photography, optical coherence tomography (OCT), OCT angiography (OCTA), fluorescein angiography (FA), and B-scan ultrasonography are several clinical imaging tools used for detecting morphological biomarkers of DR, such as vessel density percentage, microaneurysm, and retinal venular tortuosity (Flaxel et al., 2020; Midena et al., 2021b; Chen et al., 2021). Potential imaging biomarkers for DR are listed in Table 4. These computerized imaging techniques have made enormous contributions to precision therapy. In particular, OCT examines retinal layers in a noninvasive manner using automated retinal layer segmentation software (Micera et al., 2020).

**TABLE 4:** Biomarkers in imaging tools.

Biomarkers	Imaging tool	Relevance	References
Vessel density percentage	OCTA	Positive	Chen et al. (2021)
Retinal venular tortuosity	Fundus photography	Positive	Forster et al. (2021)
Fractal dimension	Fundus photography	Negative	Forster et al. (2021)
GPD	OCTA	Negative	Chen et al. (2021)
HRF	OCTA	Positive	Midena et al. (2021b)
BG-PVS severity	OCT	Positive	Choi et al. (2021)
GCL thickness	OCT	Negative	Choi et al. (2021)
FAZ	FA, OCTA	Positive	Inanc et al. (2019)
NP	UWF FA	Positive	Yu et al. (2020)
NV	UWF EA	Positive	Yu et al. (2020)

OCT, optical coherence tomography; OCTA, OCT angiography; GPD, geometric perfusion deficit; HRF, hyperreflective retinal foci; BG, basal ganglia; PVS, perivascular space; GCL, ganglion cell layer; FAZ, foveal avascular zone; FA, fluorescein angiography; NP, nonperfusion; NV, neovascularization; UWF, Ultra-wide field.

OCT, with two different scanning methods, time-domain OCT and spectral domain OCT, has been used to observe structural changes in DR and provide high-resolution imaging of the vitreoretinal interface, neurosensory retina, and subretinal space (Ishibazawa et al., 2015; Flaxel et al., 2020). OCTA is a functional extension of OCT that allows a layered view of the vascular morphology and blood flow alterations of the retina and choroid to study retinal capillary layer lesions more thoroughly (Ishibazawa et al., 2015). Although OCTA is approved by the FDA, the guidelines and indications for its use in DR screening are currently being developed. Therefore, many studies have evaluated novel imaging biomarkers based on OCTA. For example, geometric perfusion deficits are a novel OCTA biomarker based on oxygen diffusion. Compared with the vessel density percentage, geometric perfusion deficits detection is more sensitive, making analysis easier (Chen et al., 2021). Solitary, small (<30 µm) and medium level hyperreflective retinal foci, which are similar to the retinal fiber layer, may represent aggregates of activated microglial cells and serve as a biomarker of inflammation in the retina (Vujosevic et al., 2017; Midena et al., 2021b). In addition, swept-source OCTA is an improved version of OCTA that improves visualization of the vitreous and vitreoretinal interfaces, whereas technological improvements in widefield swept-source OCTA increase the field of view from 20°C to 50°C (Akiyama et al., 2018; Cui et al., 2021)

As another important diagnostic tool of DR, FA can detect primary vascular lesions (e.g., microaneurysms) and advanced vascular abnormalities (e.g., venous beading and intraretinal microvascular abnormalities) (Ishibazawa et al., 2015). However, compared to noninvasive imaging techniques, FA requires intravenous dye injection and can cause serious discomfort and stress during detection (Vujosevic et al., 2017). Furthermore, FA images are limited to two dimensions; therefore, they cannot clearly visualize the structures of layered capillary networks (Ishibazawa et al., 2015). Ultra-wide field (UWF) FA was developed based on UWF scanning laser ophthalmoscopy, which allows for the imaging of a larger area of the retina not otherwise captured (Yu et al., 2020).

## 4 Treatments

Compared to vitreoretinal surgery and laser photocoagulation, drug treatment is an emerging treatment for DR and has received increased attention in recent years.

### 4.1 Synthetic molecules

Currently, the drugs which are used in the clinical treatment of DR are all synthesized molecules. Although they cannot reverse the damage to vision caused by hyperglycemia-induced dysfunction of the retina, they do slow down the progression of DR.

In the past 10 years, anti-VEGF drugs have been used as the leading drug treatment in patients with DR. Clinical anti-VEGF drugs include ranibizumab, bevacizumab, and aflibercept (Flaxel et al., 2020; Yu H. J. et al., 2021). Ranibizumab and bevacizumab are micromolecular antibody-based drugs with a single target, whereas aflibercept is a macromolecular recombinant fusion protein that recognizes ligands of VEGF receptors 1 and 2 (Rojo Arias et al., 2020). In a recent study, aflibercept reduced the severity of retinal microvascular aberrations and significantly improved neuroretinal function (Rojo Arias et al., 2020; Yu H. J. et al., 2021). However, anti-VEGF therapy may be unsatisfactory, with some complications. First, the most serious complication of anti-VEGF injections is infectious endophthalmitis (Fu et al., 2018b). Second, long-lasting VEGF antagonism may be detrimental to the health of neurons and vascular cells because VEGF is an important neurovasculotrophic factor (Usui-Ouchi and Friedlander, 2019). Third, it may potentially increase the development of age-related macular degeneration-associated geographic atrophy which is characterized by progressive and irreversible loss of photoreceptors, RPE cells, and choriocapillaris (Usui-Ouchi and Friedlander, 2019). In addition, retinal detachment, cataract formation, and sustained elevated intraocular pressure occasionally result from VEGF therapy.

Because lipid-lowering agents have protective effects against DR progression, intravitreal corticosteroids including triamcinolone acetonide, dexamethasone, fenofibrate, omega-3 fatty acids, and statins are another important clinical drug treatment for DR (Flaxel et al., 2020; Busik, 2021). Despite their side effects of cataract progression and elevated intraocular pressure (IOP), they can be used as second-line agents for DR. However, most of the IOP caused by dexamethasone can be successfully managed using IOP-lowering medication, and no patients would require a surgery for IOP reduction (Bucolo et al., 2018). In two large randomized placebo-controlled clinical trials, fenofibrate, which is commonly used to reduce serum triacylglycerols, slowed the rate of DR progression possibly by increasing circulating hematopoietic stem/progenitor cell levels in patients with DR (Bonora et al., 2021). Furthermore, in a preclinical study, fenofibrate attenuated OS and neuroinflammation, possibly by modulating Nrf2 expression and NLRP3 inflammasome activation (Liu Q. et al., 2018).

Calcium dobesilate is the only antioxidant that is used in the clinical treatment of DR, although it is not included in clinical guidelines by the American Academy of Ophthalmology It can improve microcirculation and exert vascular-protective effects. The clinical efficacy of calcium dobesilate for DR is achieved by alleviating the high permeability of retinal vessels, which has a beneficial effect on the permeability of the BRB (Liu J. et al., 2019).

Additionally, some drugs for DR are undergoing clinical trials, such as fasudil, which is a clinically approved Rho-associated kinase inhibitor. However, owing to its short half-life, frequent repetition of intraocular injections is required, which limits further clinical development (Lebon et al., 2021). Meanwhile, we collated information on certain drugs being investigated for the treatment of DR, including anti-angiogenic, anti-vascular leakage, anti-inflammatory, anti-oxidative damage, and neuroprotective agents (Table 5). Some of these drugs play multiple roles in the treatment of DR. For example, melatonin and SZV1287 have both anti-inflammatory and antioxidant effects (Tu et al., 2021b; Tekus et al., 2021). The pathogenesis of DR is complex, therefore, multi-targeted drugs may be more effective.

**TABLE 5:** Synthetic molecules in preclinical studies.

Role	Medicine	Administraion	Mechanism	References
Anti-angiogenesis	YK-4–279	Intravitreal injection	Reduces neovascular tufts	Schafer et al. (2020)
	BT2	Intravitreal injection	Inhibitor of angiogenesis and vascular permeability by suppressing pERK-FosB/△FosB–VCAM-1 axis	Li et al. (2020b)
	UBX1967	Intravitreal injection	Targets elimination of senescent cells by inhibition of BCL-xL	Crespo-Garcia et al. (2021)
	NYY01	Intravitreal injection	Suppresses pathologic retinal neovascularization, microglial activation, and inflammatory cytokines; promotes reparative angiogenesis	Zhou et al. (2021)
	Boc-FLFLF	Subcutaneous injection	Inhibits neovessel formation	Rezzola et al. (2017)
	Linagliptin	Subcutaneous injection	Mediates GLP-1R-independent anti-angiogenic effects by inhibiting VEGFR downstream signaling	Kolibabka et al. (2018)
Prevent vascular leakage	CD5-2	Intravenous injection	Prevents vascular leakage by increasing expression of VE-cadherin, SMAD2/3 activity, and PDGF-B and reduces the activation of microglial cells	Ting et al. (2019)
	Ac-RLYE	Intravitreal injection	Prevents BRB breakdown and vascular leakage by antagonizing VEGFR-2	Park et al. (2021)
	BIRKI	Intravitreal injection	Restores RPE cell morphology and distribution, favors retinal capillary dilation, and reduces hypoxia and iBRB leakage	Lebon et al. (2021)
	β-agonists	Intraperitoneal injection	Activates PI3K/Akt signaling pathways in pericytes and attenuates pericyte loss and vascular leakage	Yun et al. (2018)
	Primaquine diphosphate	Oral	Prevents vascular leakage by maintaining endothelial integrity via ubiquitin specific protease 1 inhibition	Noh et al. (2021)
Anti-inflammatory	Semaglutide	Eye drops	Reduces glial activation, NF-κB, proinflammatory cytokines, and adhesion molecule-1; prevents RGC cell apoptosis; attenuates vascular leakage	Simo et al. (2021)
	BNN27	Intraperitoneal injection	Activates TrkA receptor and inhibits the diabetes-induced increase in p75NTR expression; decreases the activation of caspase-3, TNFa, and IL-1b; increases IL-10 and IL-4	Iban-Arias et al. (2018)
	Nimbolide	Intraperitoneal injection	Inhibits inflammation through the inhibition of the TLR4/NF-jB signaling pathway	Shu et al. (2021)
	Melatonin	Intraperitoneal injection	Inhibits inflammation and OS by enhancing the expression and activity of Sirt1	Tu et al. (2021b)
	SZV 1287	Subcutaneous injection	Inhibits AOC3; dual TRPA1/TRPV1 antagonistic activities; reduces the GFAP immunoreactivity of Müller cell processes	Tekus et al. (2021)
	Verapamil	Oral	Inhibits TLR4, TXNIP, and NLRP3-inflammasomes	Eissa et al. (2021)
	Tonabersat	Oral	Regulates assembly of NLRP3 via Connexin43 hemichannel block to reduce inflammation	Mat Nor et al. (2020)
	AMG487	Subcutaneous injection	Alleviates PDGFR-β and occludin loss and decreases the residual content of retinal albumin via the inhibition of OS and ER stress and activation of p38	Wang et al. (2019)
Neuroprotection	PF-05231023	Intraperitoneal injection	Reduces inflammatory marker IL1b mRNA levels; activates the Akt-Nrf2 pathway in photoreceptors	Fu et al. (2018b)
	rhNGF	Intravitreal injection/eye drop	Recovers optic nerve crush-induced RGC degeneration by reversing the proNGF/p75NTR increase and TrkA receptors activation	Mesentier-Louro et al. (2019)
	Liraglutide	Intravitreal injection	Arrests hyperphosphorylated tau-triggered retinal neurodegeneration via activation of GLP-1R/Akt/GSK3β signaling	Shu et al. (2019)
	Rapamycin	Intraperitoneal injection	Prolongs autophagy activation and improves RGC survival	Russo et al. (2018)
	hydroxytyrosol	Oral	Decreases peroxynitrite production; antiplatelet effect; protects endothelial prostacyclin production	Gonzalez-Correa et al. (2018)

BCL-xL, B cell lymphoma-xL; Boc-FLFLF, Boc-Phe-Leu-Phe-Leu-Phe; GLP-1R, glucagon-like peptide 1 receptor; VEGFR, vascular endothelial growth factor receptor; PDGF, platelet-derived growth factor; Ac-RLYE, N-acetylated Arg-Leu-Tyr-Glu; PI3K, phosphoinositide 3-kinase; TLR4, toll-like-receptor-4; TXNIP, thioredoxin-interacting protein; NLRP3, nucleotide-binding domain-like receptor protein-3; p75NTR, neurotrophin receptor p75; TrkA, tropomyosin-related kinase A; IL, interleukin; TRPA1/TRPV1, transient receptor potential ankyrin 1 and vanilloid 1; GFAP, glial fibrillary acidic protein; Keap1, Kelch-like erythroid-cell-derived protein with CNC homology-associated protein 1; BIRKI, Boehringer Ingelheim Rho kinase inhibitor; UPARANT, Ac-L-Arg-Aib-L-Arg-L-Cα(Me)Phe-NH2 tetrapeptide; Sirt1, silent information regulator factor 2-related enzyme 1; rhNGF, recombinant human nerve growth factor; GSK3β, glucogen synthase kinase 3

### 4.2 Natural molecules

It is well known that natural materials have a long history with rich clinical experience in preventing and treating diseases, and these materials include plants, animals, microbes, and minerals. Natural molecules extracted from natural materials are important sources of drugs, and plants are the most commonly used natural materials. Additionally, with the development in society, people are increasingly becoming concerned about their health, and returning back to nature has become a trendy idea. Therefore, natural molecules have a great potential value. Recently, an increasing number of experimental studies have been conducted on the mechanism of natural molecules in the prevention and treatment of DR. We have compiled certain natural molecules (in preclinical studies) used for the treatment of DR in recent years (Table 6). Moreover, marine organisms serve as an emerging source of novel bioactive compounds and can be developed for the treatment of DR. For example, fucoxanthin extracted from seaweed has a protective effect on the retina by increasing catalase and reducing OS (Chiang et al., 2020).

**TABLE 6:** Natural molecules.

Medicine	Major source	Mechanism	References
Asiatic acid	*Centella asiatica* (L.) Urban	Ameliorates early DR by regulating microglia polarization by the TLR4/MyD88/NF-κB p65 pathway	Fang et al. (2021)
Berberine	*Coptis chinensis* Franch	Suppresses AGE formation through TLR4/STAT3/VEGF signaling pathway in ECs	Wang et al. (2021)
Caffeine	*Coffea arabica* L	Counteracts inflammation	Conti et al. (2021)
Chlorogenic acid	*Lonicera japonica* Thunb	Alleviates BRB injury by reducing microglia-initiated inflammation; prevents TNF-α-induced EMT and oxidative injury by inducing activation of Nrf2	Mei et al. (2018)
Curcumin	*Curcuma longa* L	Suppresses oxidative stress to protect ECs via regulation of ROS/NF-κB pathway; Protect RPEs via ERK1/2-mediated activation of the Nrf2/HO-1 pathway	(Platania et al., 2018; Bucolo et al., 2019; Huang et al., 2021)
Dihydrotanshinone	*Salvia miltiorrhiza* Bunge	Preserves BRB integrity from high glucose/BzATP damage; inhibits inflammation by acting on TLR-4	Fresta et al. (2020a)
Ginsenoside Rg1	*Panax ginseng* C. A. Mey	Prevents hyperphosphorylated tau-induced synaptic neurodegeneration of RGCs by activating IRS-1/Akt/GSK3β signaling	Ying et al. (2019)
Gypenoside XVII	*Panax poseudogindeng* Wall	Decreases apoptosis and increases autophagy in Müller cells	Luo et al. (2021)
Geniposide	*Gardenia jasminoides* Ellis	Alleviates oxidative stress and inflammation through the Nrf2 signaling pathway via GLP-1R	Tu et al. (2021a)
Lutein	Widely found in plants	Inhibits the growth of RPE cells and protect them against oxidative stress-induced cell loss	Gong et al. (2017)
Lycopene	*Lycopersicon esculentum* Mill	Inhibits the growth of RPE cells and protects them against oxidative stress-induced cell loss	Gong et al. (2017)
Kaempferol	*Kaempferia galanga* L	Protects RPE cells against oxidative stress damage and apoptosis	Du et al. (2018)
Quercetin	*Pinaceace*	Induces HO-1 expression	Chai et al. (2021)
Resveratrol	*Veratrum album*	Counteracts NOX-mediated EMT in ECs via inhibition of PKC	Giordo et al. (2021)
Sulforaphane	Cruciferae	May delay photoreceptor degeneration may via inhibition of ER stress, inflammation, and Txnip expression through the activation of the AMPK pathway	Lv et al. (2020)

STAT3, signal transducer and activator of transcription 3; TNF-α, tumor necrosis factor-α; EMT, endothelial-mesenchymal transition; ERK1/2, extracellular signal regulated kinases 1 and 2; HO-1, heme oxygenase-1; BzATP, 2'(3')-O-(4-Benzoylbenzoyl)adenosine-5'-triphosphate; IRS-1, insulin receptor substrates 1; PKC, protein kinase C.

### 4.3 Administration

The administration routes for synthesized molecules in preclinical studies include injection, oral, and eye drops (see Table 5). Among the injectable routes, intravitreal injection is the most commonly used clinical method for the treatment of DR. However, compared to intravitreal injection, subcutaneous injection, intraperitoneal injection, and intravenous injection are more suitable as they are noninvasive to the eyes, although they are usually only used in animal models of DR at present. Additionally, pharmacokinetic and pharmacodynamics changes with aging also represent an important aspect that needs to be considered (Leley et al., 2021).

## 5 Perspective

In recent years, the definition of DR has transformed from microangiopathy to highly tissue-specific neurovascular complication, and neurodegeneration in DR has become a hot research topic. However, the pathological mechanisms of DR are complex and diverse, and several potential mechanisms require further in-depth research and investigation. The spatiotemporal correlations between the currently known pathological mechanisms have not been integrally studied. Therefore, this could be a potentially important research direction for future studies. A clear sequence of DR pathogenesis in time and space could facilitate the identification of the progress and the therapeutic targets of DR.

Understanding the pathological mechanism of DR is the basis for the development of new biomarkers of this disease. The lack of effective diagnostic biomarkers for DR can lead to unsatisfactory curative treatments, and the identification of biomarkers is crucial for uncovering the underlying mechanisms of DR and making a clear classification in clinical diagnosis. More importantly, research on potential drug targets is based on clear pathogenesis and effective biomarkers. Currently, the search for biomarkers and therapeutic interventions for DR is focused on the treatment of late phases of the disease. Therefore, biomarkers and drugs for early DR (including neuropathy and NPDR) will be the key for future research. In addition, combinatorial biomarkers are worth considering, as their sensitivity is significantly higher than that of individual biomarkers.

Currently, although there are drugs already approved for DR treatment, they are not completely effective in curing DR, and most of them only decelerate disease progress. Thus, the development of new drugs remains crucial. However, pathological mechanisms of DR are complicated, and combination drug therapy with versatile targets is perhaps more effective for DR treatment.
